# Sex Differences in Kappa Opioid Receptor Function and Their Potential Impact on Addiction

**DOI:** 10.3389/fnins.2015.00466

**Published:** 2015-12-16

**Authors:** Elena H. Chartoff, Maria Mavrikaki

**Affiliations:** Department of Psychiatry, Harvard Medical School, McLean HospitalBelmont, MA, USA

**Keywords:** female, depression, drug withdrawal, antinociception, estrogens, dopamine, analgesia

## Abstract

Behavioral, biological, and social sequelae that lead to drug addiction differ between men and women. Our efforts to understand addiction on a mechanistic level must include studies in both males and females. Stress, anxiety, and depression are tightly linked to addiction, and whether they precede or result from compulsive drug use depends on many factors, including biological sex. The neuropeptide dynorphin (DYN), an endogenous ligand at kappa opioid receptors (KORs), is necessary for stress-induced aversive states and is upregulated in the brain after chronic exposure to drugs of abuse. KOR agonists produce signs of anxiety, fear, and depression in laboratory animals and humans, findings that have led to the hypothesis that drug withdrawal-induced DYN release is instrumental in negative reinforcement processes that drive addiction. However, these studies were almost exclusively conducted in males. Only recently is evidence available that there are sex differences in the effects of KOR activation on affective state. This review focuses on sex differences in DYN and KOR systems and how these might contribute to sex differences in addictive behavior. Much of what is known about how biological sex influences KOR systems is from research on pain systems. The basic molecular and genetic mechanisms that have been discovered to underlie sex differences in KOR function in pain systems may apply to sex differences in KOR function in reward systems. Our goals are to discuss the current state of knowledge on how biological sex contributes to KOR function in the context of pain, mood, and addiction and to explore potential mechanisms for sex differences in KOR function. We will highlight evidence that the function of DYN-KOR systems is influenced in a sex-dependent manner by: polymorphisms in the prodynorphin (pDYN) gene, genetic linkage with the melanocortin-1 receptor (MC1R), heterodimerization of KORs and mu opioid receptors (MORs), and gonadal hormones. Finally, we identify several gaps in our understanding of “if” and “how” DYN and KORs modulate addictive behavior in a sex-dependent manner. Future work may address these gaps by building on the mechanistic studies outlined in this review. Ultimately this will enable the development of novel and effective addiction treatments tailored to either males or females.

There is increasing evidence in humans and laboratory animals for biologically-based sex differences in every phase of drug addiction: acute reinforcing effects, transition from occasional to compulsive use, withdrawal-associated negative affective states, craving, and relapse (Griffin et al., 1989; McKay et al., 1996; Lynch and Taylor, 2004; Lynch, 2006; Fox and Sinha, 2009; Greenfield et al., 2010). In general, as an individual moves from impulsive to compulsive drug use, it is thought that a shift occurs such that negative reinforcement dominates over positive reinforcement as the driving force for motivated behavior (Koob, 2008). Although the qualities and magnitude of drug withdrawal syndromes vary among different class of drugs, withdrawal exacerbates reward deficits that contribute to negative reinforcement and contributes to drug craving and relapse (Kenny, 2007; Koob and Le Moal, 2008). Furthermore, repeated drug exposure produces long-lasting neural adaptations that sensitize the organism to drugs and drug-associated cues. Thus, in humans, motivational focus narrows to drug seeking at the expense of natural reward seeking. Consistent with negative reinforcement mechanisms of addiction, one consistent finding in human studies is that drug-dependent women express greater negative emotional states such as stress and depression (Griffin et al., 1989), and these are more likely to trigger craving and relapse in women than men (McKay et al., 1996; Fox and Sinha, 2009). The predominance of comorbid mood disorders in female drug addicts likely arises from preexisting psychopathologies. In the population as a whole, women are twice as likely as men to suffer from anxiety and depression (Kessler, 2003). Although women are more likely to seek treatment, they experience longer episodes of depression and lower rates of recovery (Weissman and Olfson, 1995). Thus, sex differences in stress and reward pathways may explain why women are more vulnerable than men to the negative consequences of drug dependence (Becker et al., 2012). Paradoxically, the prevalence of drug dependence is generally reported to be higher in adult men compared to women (Carroll et al., 2004). Interestingly, more recent epidemiological studies indicate a narrowing in this gender gap (Substance Abuse and Mental Health Services Administration, 2014)—particularly in adolescents. This may reflect changing sociocultural patterns, rather than biology.

Chronic exposure to drugs of abuse elicits neuroadaptations in stress and reward pathways, including enhanced synthesis and release of dynorphin (DYN)—an endogenous ligand at kappa opioid receptors (KORs; Chavkin et al., 1982). Several studies have shown that psychostimulant-induced increases in DYN signaling are coincident with the emergence of depressive-like effects including anhedonia and anxiety (Hurd and Herkenham, 1993; Cole et al., 1995; Shippenberg et al., 2007). However, relatively little is known about the role of DYN and KORs in motivated behavior or drug withdrawal-induced negative affective states in females. Recent work from our lab shows that female rats are less sensitive than males to the depressive-like effects of a KOR agonist (Russell et al., 2014) raising the possibility that the contribution of KORs to negative reinforcement processes differs between the sexes. The purpose of this review is to discuss the current state of knowledge on the regulation and role of DYN and KORs within neural circuits important for emotional processing, with particular emphasis on putative mechanistic underpinnings of these sex differences. Given the nascent state of this field, we will also discuss more established data on sex differences in the role of KORs in nociception with the goal of identifying overlapping mechanisms as well as gaps in our knowledge.

## Dynorphin and kappa opioid receptors (KORs)

Dynorphins are a class of opioid peptides that arise from the precursor protein prodynorphin (pDYN) and act as endogenous ligands at the KOR (Chavkin and Goldstein, 1981). DYN and KORs are found throughout the central and peripheral nervous system (Mansour et al., 1988, 1995; Le Merrer et al., 2009), where they modulate a diverse set of physiological outputs including nociception, hypothermia, and water diuresis (Vonvoigtlander et al., 1983), as well as motivated behavior and affective states (Bruchas et al., 2010; Knoll and Carlezon, 2010). Protease cleavage of pDYN during processing releases multiple active peptides selective for the KOR including DYN A_1−8 &__1−17_, DYN B, and α/β-neoendorphins (Chavkin, 2013). The human pDYN gene contains four exons: exons 1 and 2 encode the 5′ untranslated region (UTR), exon 3 encodes a signal peptide, and exon 4 encodes the DYN peptides (Horikawa et al., 1983). The pDYN gene contains a non-canonical activating protein 1 (AP-1)-Iike site in its promoter region, which is a target for Fos/Jun trans-activation. In addition, the pDYN promoter has been shown to contain three cAMP response elements (CRE) elements, which are targets for CRE binding protein (CREB)—mediated transcriptional activation (Douglass et al., 1989, 1994; Cole et al., 1995).

Dynorphins are contained in large dense core vesicles and primarily released in a calcium-dependent manner (Chavkin et al., 1983). They can be released from nerve terminals to cause presynaptic autoinhibition or from dendrites to cause retrograde inhibition of afferents (for review, see Simmons and Chavkin, 1996). Activation of KORs leads to a variety of effects on intracellular signaling pathways, including decreased cAMP production, increased K^+^ and decreased Ca^2+^ channel conductance, and activation of extracellular signal-regulated kinase (ERK) pathways (Grudt and Williams, 1995; Law et al., 2000; Bruchas et al., 2008; Potter et al., 2011). After sustained KOR activation, receptor desensitization can occur in which G-protein coupled receptor kinase 3 (GRK3) phosphorylates the KOR at serine 369 (KOR-P) (Liu-Chen, 2004). This initiates arrestin-dependent receptor internalization and creates a receptor-protein complex that recruits additional second messenger signaling systems (Bruchas and Chavkin, 2010).

There is evidence in adults that gonadal hormones can regulate the expression and release of dynorphins in spinal cord, hypothalamic nuclei and in hippocampus (Gintzler et al., 2008; Gottsch et al., 2009; Torres-Reveron et al., 2009). These effects have not yet been tested for a connection to drug reward and addictive behavior. To date, the little information available on sex differences in KOR systems comes primarily from studies on KOR-mediated antinociception (Rasakham and Liu-Chen, 2011).

## Influence of sex on brain and behavior

Sex differences in behavior can be classified into three main types (McCarthy and Arnold, 2011). First, behavioral endpoints can exist in either “male” or “female” forms or be present in one sex and absent in the other. Since both men and women can become addicted to drugs, there is not an absolute sexual dimorphism in addiction *per se*. However, it is possible that drugs of abuse produce a specific behavioral or neurochemical outcome in only one sex. Second, behavioral endpoints can exist on a spectrum in which the average behavior is different in males and females (e.g., pain thresholds and analgesic efficacy of opioid painkillers). This is the most commonly observed type of sex difference in drug addiction research. A challenge for neuroscientists is ascertaining whether such differences are socially or biologically based. Third, behavioral endpoints can be the same in males and females, but the underlying neural mechanisms that produce the behavior can be different (e.g., certain stress responses). This type of sex difference often goes undetected or unreported, since there are no overt behavioral differences between males and females. But sex differences in the mechanisms that lead to addictive behavior could profoundly influence the way in which addiction is treated.

The X and Y sex chromosomes are the origin of all biological sex differences (McCarthy and Arnold, 2011). Expression of the Y-linked gene, *Sry*, leads to testes development in males, which subsequently secrete testosterone (Goodfellow and Lovell-Badge, 1993; Becker et al., 2005). In the absence of *Sry*, ovaries develop, which secrete estrogen and progesterone. The timing and mechanisms by which the sex chromosomes influence sexual dimorphism vary in three primary ways. First, there are direct effects of genes expressed on X and Y chromosomes on brain and somatic tissues. This can be through unique gene expression or sex-dependent expression levels of the same genes. For example, *Sry* is expressed in midbrain dopamine-containing cells and has direct male-specific effects (Dewing et al., 2006). The gene *Xist* is expressed from one of the two X chromosomes in non-germline cells in females and is responsible for inactivation of that chromosome, thus insuring that gene expression between males and females is fairly consistent. However, some genes escape X inactivation in a tissue-specific manner. For example, pDYN gene expression is higher in the striatum of XX mice, independent of gonadal hormones, suggesting that inactivation is incomplete, at least in this brain region (Chen et al., 2009). The second way that sex chromosomes influence sexual dimorphism is through organizational effects of gonadal hormones during fetal and neonatal periods. The effects of circulating gonadal hormones on brain development in humans are greatest during weeks 8 through 24 of gestation (McCarthy, 2010). During this critical period, neural structures are still being established in the fetus. At this time, male fetuses secrete over 2.5 times more testosterone than females (Auyeung et al., 2013). In males, circulating testosterone is aromatized to estradiol in the central nervous system. There it masculinizes and defeminizes the brain, modulating synapses and shaping the formation of brain regions (McCarthy and Arnold, 2011). For example, organizational processes sexualize brain structures such as the preoptic area and the suprachiasmatic nucleus of the hypothalamus, the basolateral amygdala (BLA), the bed nucleus of the stria terminalis (BNST), and the corpus callosum (Collaer and Hines, 1995). Of particular importance to addiction and mood disorders are the amygdala and the BNST (Bruchas et al., 2010; Knoll and Carlezon, 2010). Third, there are activational effects of circulating gonadal hormones during and after puberty (McCarthy et al., 2012) Fluctuations in hormone secretion may affect behaviors on a short-term scale, but are quickly mitigated after steroid concentrations return to baseline (McCarthy et al., 2012).

**Figure 1:**
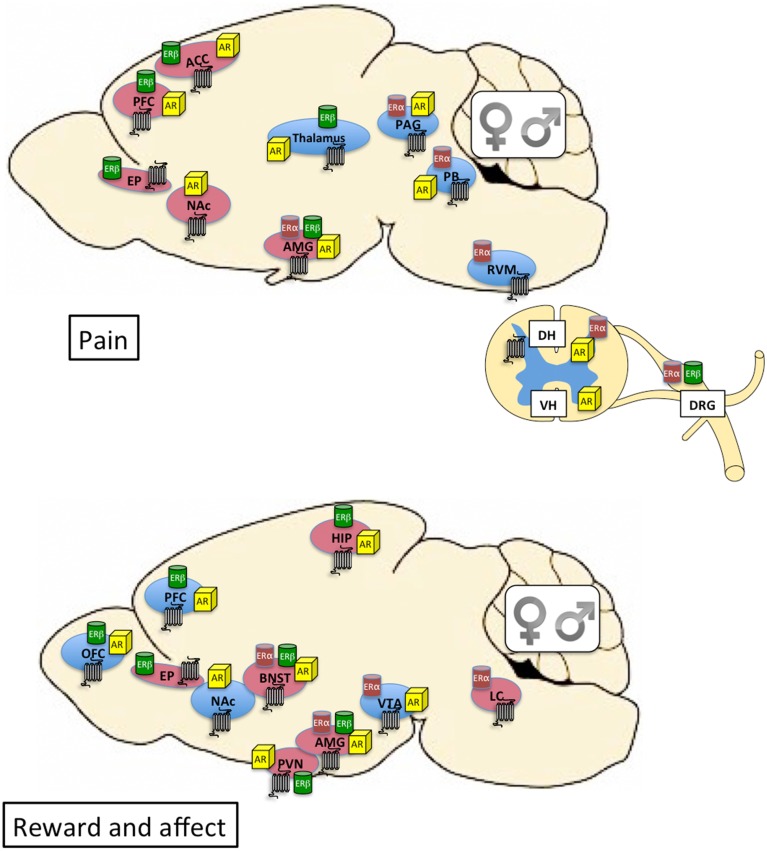
**Top:** Distribution of kappa opioid (7-transmembrane receptor), estrogen, and androgen receptors within neural circuits involved in pain. Brain regions shaded in blue are primarily associated with sensory dimensions of pain whereas those shaded in red are primarily associated with affective dimensions of pain. There is crosstalk between the various brain regions (see Cahill et al., 2014 for review). **Bottom:** Distribution of kappa opioid, estrogen, and androgen receptors within neural circuits involved in affective state and motivated behavior. Brain regions shaded in blue are primarily associated with the mesocorticolimbic dopamine system and reward processing. Brain regions shaded in red are primarily associated with stress and anxiety (negative affective states). Expression of KORs, ERs, and ARs is qualitatively similar between males and females. The final impact of KOR activation on neuronal function and behavior depends on the cellular localization of the receptor (e.g., pre- or post-synaptic) and the projection target of the KOR-expressing neuron. Currently this level of detail is relatively unexplored in male vs. female brain. Receptor expression in this figure is based on evidence of overlapping mRNA expression and receptor binding: indicated brain regions are thought to synthesize and express the indicated receptors (Morris and Herz, 1987; Mansour et al., 1988, 1995; Besse et al., 1990; Simerly et al., 1990; Arvidsson et al., 1995; Lumbroso et al., 1996; Shughrue et al., 1997; Laflamme et al., 1998; Slowe et al., 1999; Papka et al., 2001; Hamson et al., 2004; Harris et al., 2004; Vanderhorst et al., 2005; Loyd and Murphy, 2008; Le Merrer et al., 2009; Feng et al., 2010; Rasakham and Liu-Chen, 2011). ACC, anterior cingulate cortex; AMG, amygdala; AR, androgen receptor; BNST, bed nucleus of the stria terminalis; DH, dorsal horn of spinal cord; DRG, dorsal root ganglia; EP, endopiriform cortex; ER, estrogen receptor; HIP, hippocampus; LC, locus coeruleus; NAc, nucleus accumbens; OFC, orbitofrontal cortex; PAG, periaqueductal gray; PB, parabrachial nucleus; PFC, prefrontal cortex; PVN, paraventricular nucleus of the hypothalamus; RVM, rostral ventral medulla; VH, ventral horn of the spinal cord; VTA, ventral tegmental area.

The steroid hormones testosterone, estrogen, and progesterone activate androgen (AR), estrogen α and β (ERα and ERβ), and progesterone (PR) receptors, respectively. These act primarily as nuclear transcription factor receptors capable of directly interacting with DNA at specific response elements and modulating gene transcription (Beato, 1989). It is also clear, from more recent research, that steroid hormones act rapidly on membrane bound receptors to activate signal transduction pathways with broad effects on cellular physiology (Balthazart and Ball, 2006). Indeed, a new membrane-bound G protein-coupled estrogen receptor (GPER-1/GPR30) has been identified (Revankar et al., 2005) and implicated in the non-genomic effects of estrogen (Elkabes and Nicot, 2014). Both males and females can produce testosterone and estrogen, although the divergent effects of these steroid hormones are mediated by sex differences in their levels, distribution and metabolism, as well as the levels and distribution of their cognate receptors (McCarthy and Arnold, 2011). Although there are many hormone-sensitive areas of the brain, of particular importance to drug addiction is the presence of steroid hormone receptors within neural circuits associated with mood regulation and stress sensitivity, such as the mesocorticolimbic system, the extended amygdala, and the hypothalamic pituitary adrenal (HPA) axis (see Figure 1). As such, it is likely that both organizational and activational effects of gonadal hormones within these neural circuits contribute to sex differences in motivated behavior and affective states.

## Role of KOR systems in pain

### Human

Sex differences have been reported in the perception of pain (Gear et al., 1996; Fillingim, 2000; Sibille et al., 2011) and in the efficacy of opioid analgesics to ameliorate pain (Fillingim et al., 2004; Zacny and Beckman, 2004; Bodnar and Kest, 2010; Rasakham and Liu-Chen, 2011). In general, women tend to demonstrate lower pain thresholds, less pain tolerance and higher evoked pain intensity (Gear et al., 1996; Riley et al., 1998; Sibille et al., 2011) as well as report higher pain ratings (Zacny and Beckman, 2004). However, there is significant variability between and within studies, the effect sizes are often very small, and sex differences only exist for some types of pain measures (Berkley, 1997; Wiesenfeld-Hallin, 2005).

Similar to MOR agonists such as endorphins and morphine, DYN and exogenous KOR agonists have antinociceptive properties (Han and Xie, 1982, 1984) but with a much lower abuse potential (Fraser and Rosenberg, 1964). Just as sex differences in the perception of pain are complex, the analgesic effects of opioids in men and women depend on the specific type of pain and the opioid analgesic. Mixed KOR/MOR partial agonists including pentazocine, nalbuphine, and butorphanol have been shown to produce greater analgesia in women compared to men in post-operative dental surgery (Gear et al., 1996, 1999). Similarly, the analgesic effect of pentazocine has been shown to be more robust in women for ischemic and thermal pain (Mogil et al., 2003). In contrast, Fillingim et al. (2004) reported no sex differences in the analgesic effects of pentazocine in models of heat, ischemic and pressure pain. There are also numerous examples in which opioid analgesics have greater effects in men. The partial MOR/KOR agonist butorphanol produces higher analgesic ratings in men in the cold-water stimulus pain assay (Zacny and Beckman, 2004), although other studies suggest no sex differences in the analgesic effects of either the MOR agonist morphine or butorphanol (Sibille et al., 2011). Importantly, clearance of butorphanol has been shown to be lower in women compared to men, raising the possibility that pharmacokinetic differences between men and women are a factor and should be considered in dose determinations (Shyu et al., 1994). Taken together, it is almost impossible to draw sweeping conclusions about how sex impacts pain and analgesia. Rather, the findings to date highlight the importance of studying each facet of analgesia in both men and women and suggest that genetic variability plays a major role.

### Animals

According to a recent analysis, approximately 80% of the animals studies published in the journal “Pain” in the last decade have been done in males and only 4% of those studies were designed to test pain perception in both sexes (Bodnar and Kest, 2010). Animal studies examining sex differences in pain or analgesic sensitivity have revealed that the presence and direction of sex differences are dependent on the strain chosen for study (Mogil et al., 2000). The interactions between sex and genetics on pain response are still relatively unexplored, but identification of relevant genes should facilitate development of more effective analgesics for both men and women.

Interestingly, animal studies tend to demonstrate more robust analgesic effects of KOR ligands in males compared to females, whereas the opposite is generally true in human studies (see Rasakham and Liu-Chen, 2011). For example, the selective KOR agonist U50,488 has more robust analgesic effects in male rats and mice compared to females in the tail flick assay (Kavaliers and Innes, 1987; Barrett et al., 2002; Mogil et al., 2003; Sternberg et al., 2004a; Stoffel et al., 2005), although this does not hold for every strain (Rasakham and Liu-Chen, 2011). Reproductive hormones such as testosterone and estradiol do not seem to significantly modulate sensitivity to the analgesic effects of opioids under many—but not all—conditions (Stoffel et al., 2005). The sex differences in the antinociceptive effects of KOR agonists have been suggested to be primarily spinal rather than supraspinal dependent (Craft and Bernal, 2001).

### Neuroanatomy of KORs related to pain

DYN and KORs in the peripheral and central nervous system play major roles in the sensory and affective components of pain (Cahill et al., 2014). As such, the neural circuits sub serving pain and affect are at least partially overlapping (see Figure 1). Chronic pain stimuli lead to sustained DYN release in the spinal cord, which is thought to be a compensatory analgesic response (Iadarola et al., 1988; Cahill et al., 2014). Thus, it is thought that analgesic effects of KOR activation are due in part to DYN and KOR localization in the spinal cord and brain stem, where they act to inhibit pain pathways (Millan et al., 1989; Watkins et al., 1992; Simonin et al., 1995; Yaksh, 1997; Cahill et al., 2014). In the spinal cord, KORs are localized pre- and post-synaptically (Ji et al., 1995): a proportion are expressed in the superficial dorsal horn of the spinal cord (Gouardères et al., 1986; Maekawa et al., 1994; Mansour et al., 1994b, 1996; Arvidsson et al., 1995) and a proportion (~50%) on sensory afferents from the dorsal root ganglion (Besse et al., 1990; Maekawa et al., 1994; Mansour et al., 1994b, 1996; Arvidsson et al., 1995). KORs are also co-expressed with other opioid receptors (mu and delta) on primary sensory neurons. In rat and rabbit, the highest levels of DYN immunoreactivity have been observed in the dorsal part of the spinal cord, and the lowest levels in the dorsal root ganglia (Botticelli et al., 1981). KOR agonists have been shown to modulate excitatory transmission in substantia gelatinosa neurons of the rat spinal cord via inhibition of AMPA-mediated neurotransmission (Randic et al., 1995).

In addition to spinal actions of DYN (Han and Xie, 1982), there is evidence supporting analgesic KOR effects at supraspinal sites (Cahill et al., 2014) including parietal cortex and amygdala (Abraham et al., 2000; Narita et al., 2006). The rostral ventromedial medulla (RVM) has also been shown to be a crucial site for the supraspinal antinociceptive actions of opioids. In particular, the nucleus raphe magnus forms a component of a descending inhibitory network that modulates nociceptive neurotransmission at the level of the spinal cord dorsal horn (Marinelli et al., 2002). This nucleus projects to the spinal cord and approximately 20% of the neurons respond to KOR ligands (Marinelli et al., 2002). Pain transmission pathways from the periphery to the CNS involve projections from the medulla to the midbrain and more anterior structures (Al-Hasani and Bruchas, 2011), which overlap with structures involved in KOR effects on affective state (see Figure 1).

## Role of KOR systems in affective state

### Human

Based on the hypothesis that KOR agonists could be utilized as non-addictive analgesics, a major effort was initiated to develop and test KOR-selective ligands in pain models. However, patients (only males were included in these early studies) reported aversive and depressive signs (including dysphoria) after treatment with non-selective KOR agonists such as cyclazocine, spiradoline, and the more selective KOR agonist MR2033 (Pfeiffer et al., 1986; Chappell et al., 1993). Interestingly, pentazocine, a relatively specific KOR partial agonist/MOR antagonist (Zhu et al., 1997) is still used for obstetrical pain, perhaps because females are less sensitive to the aversive effects of KOR activation (Russell et al., 2014). Indeed, a case report describes antidepressant effects of the naturally occurring hallucinogen *Salvia divinorum*, a rare member of the mint family in which the active ingredient is the highly selective and potent KOR agonist, salvinorin A, in a woman with intractable depression (Hanes, 2001). In contrast to this, a retrospective survey of recreational *Salvia* users reported similar levels of drug-induced anxiety in men and women (Gonzalez et al., 2006). Given the negative effects of KOR agonists on mood, drug development efforts for KOR ligands as analgesics were largely halted. However, the information gleaned from these studies has been instrumental in our understanding of the role of DYN/KORs in affective state and has led to novel hypotheses about the clinical utility of KOR-selective compounds (Carlezon et al., 2009).

One such hypothesis is that KOR-mediated reductions in hyperdopaminergia may reduce symptoms of mania in people with bipolar disorder. This was tested using pentazocine in a small open-label study in 10 patients (7 male and 3 female) with mania. Pentazocine reduced manic symptoms transiently but substantially and significantly in each subject without causing notable sedation or affecting psychotic symptoms (Cohen and Murphy, 2008).

A second hypothesis that has gained traction is that KOR antagonists may reduce depressive-like symptoms that characterize numerous psychiatric disorders including major depressive disorder (MDD), post-traumatic stress disorder (PTSD), and drug addiction. In support of this, buprenorphine, a partial MOR agonist/ KOR antagonist, has been shown to have antidepressant efficacy in MDD in observational studies (Emrich et al., 1982; Walsh et al., 1994, 1995; Bodkin et al., 1995; Cowan, 2003; Nyhuis et al., 2008; Karp et al., 2014) and to reduce depressive symptoms in heroin addicted patients who were depressed at intake (Kosten et al., 1990). Amazingly, these studies were conducted only in males or, when females were included, no information is provided on the contribution of gender to the results. More recently, attempts have been made to isolate the KOR antagonist properties of buprenorphine by co-administering MOR antagonists. Rothman and colleagues hypothesized that a KOR antagonist would alleviate dysphoria observed in the protracted opiate withdrawal syndrome and as such reduce rates of relapse in abstinent opioid-dependent patients. A combination of buprenorphine and the MOR antagonist naltrexone has been shown to produce a substantial reduction in cocaine and opiate use (Rothman et al., 2000; Gerra et al., 2006). Perhaps the most compelling clinical data supporting antidepressant effects of KOR antagonists come from a recent study in which a combination of buprenorphine and the potent MOR antagonist samidorphan administered to subjects with treatment resistant MDD resulted in significant and substantial antidepressant activity without addictive potential (Ehrich et al., 2015).

At this point there is little conclusive evidence regarding sex differences in the direct role of DYN and KOR systems in drug addiction in humans. However, there is increasing evidence for sex differences in the role of KORs in mediating stress responses and affective states in preclinical animal models, which may help us understand, on a mechanistic level, why addiction is experienced differently in men and women.

### Animals

In males, activation of KORs produces depressive-like and anxiogenic behaviors (Bals-Kubik et al., 1989; Todtenkopf et al., 2004; Carlezon et al., 2006; Tomasiewicz et al., 2008; Ebner et al., 2010; Knoll et al., 2011; Muschamp et al., 2011), encodes the dysphoric component of stress (Land et al., 2008; Bruchas et al., 2010), and can promote drug-seeking behavior (Negus, 2004; McLaughlin et al., 2006; Redila and Chavkin, 2008; Schindler et al., 2010). Likewise, KOR blockade has antidepressant and anxiolytic effects (Pliakas et al., 2001; Newton et al., 2002; Mague et al., 2003; Knoll et al., 2007), attenuates aversive states associated with cocaine withdrawal (Chartoff et al., 2012), and attenuates drug-seeking behavior (Beardsley et al., 2005; Carey et al., 2007; Walker and Koob, 2007; Redila and Chavkin, 2008; Land et al., 2009; Wee et al., 2009; Bruchas et al., 2010). These findings are consistent with observations that KOR agonists produce dysphoric and depressive-like states in men (Pfeiffer et al., 1986).

We recently demonstrated that female rats are less sensitive than males to the depressive-like effects of the KOR agonist U50,488 (Russell et al., 2014) using intracranial self-stimulation (ICSS), an operant behavior that is sensitive to increases and decreases in reward function (Carlezon and Chartoff, 2007). This effect is independent of circulating gonadal hormones, as gonadectomy did not alter responses to U50,488 (Russell et al., 2014). Cellular mapping showed U50,488-induced c-Fos activation to be greater in corticotropin releasing factor (CRF)-containing neurons of the paraventricular nucleus of the hypothalamus (PVN) and in non-CRF-containing neurons of the BNST oval nucleus in females compared to males (Russell et al., 2014). In an earlier study, it was shown that female rats take significantly longer than males to discriminate the KOR agonist U69,593 from vehicle using an FR-10 schedule of food reinforcement (Craft et al., 1998). The ED50 for U69,593 discrimination was significantly higher in females, and the peak and offset for U69,593 occurred earlier in females compare to the males (Craft et al., 1998). In contrast, Robles et al. (2014) demonstrated in the California mouse that females formed conditioned place aversions to a low, but place preferences to a high, dose of U50,488. However, males were insensitive to the low and formed conditioned place aversions to the high dose of U50,488. Together these findings highlight how species and behavioral paradigm play a major role in the function of KORs.

In males, co-administration of KOR agonists with cocaine has been shown to prevent the development of locomotor sensitization to cocaine as well as cocaine-induced conditioned place preferences (Heidbreder et al., 1995; Shippenberg and Rea, 1997). However, there is emerging evidence that prolonged or prior exposure to KOR agonists can potentiate the effects of cocaine. For example, exposure to KOR agonists can increase the locomotor stimulant effects of psychostimulants as well as stimulated dopamine release in striatal regions, depending on the timing and context of KOR agonist exposure (Heidbreder et al., 1998; Fuentealba et al., 2006, 2007; Chartoff et al., 2008). Continuous treatment of rhesus monkeys with the KOR agonist U50,488 increases cocaine choice (Negus, 2004), suggesting a KOR-mediated increase in the relative reinforcing effects of cocaine. Pretreatment with KOR agonists can potentiate cocaine-induced place preferences (McLaughlin et al., 2006) and brain stimulation reward (Chartoff et al., 2015), and activation of KORs can reinstate cocaine seeking (Redila and Chavkin, 2008). One explanation for KOR-mediated potentiation of cocaine reward that has been proposed (McLaughlin et al., 2006) is that activation of KORs prior to administration of cocaine produces a *dysphoric* state that enhances the rewarding properties of cocaine. This raises the possibility that, in females, KOR activation would fail to potentiate reward-related effects of drugs of abuse. This has yet to be directly tested.

Consistent with idea that KOR activation can enhance psychostimulant effects in males but not females, it has been reported that pretreatment with the non-selective KOR agonist spiradoline potentiates acute cocaine-induced locomotor activity in male, but not female, mice (Sershen et al., 1998). Further *in vitro* analyses indicated that the ability of spiradoline to inhibit NMDA-evoked dopamine release in striatal slices was lower in female compared to male tissue (Sershen et al., 1998). This is consistent with the general concept that the net change (delta) in dopamine signaling determines behavioral responses to cocaine (Ehrich et al., 2014; Chartoff et al., 2015). In guinea pigs, which have KOR expression levels and distributions quite similar to humans, the effects of the KOR agonist U50,488 on basal measures of body posture and analgesia were shown to be consistently lower in females compared to males (Wang et al., 2011). KOR effects on basal locomotor activity did not differ between males and females, whereas acute cocaine-induced locomotor activity was substantially higher in females compared to males. In the same study, U50,488 produced greater decreases in cocaine-induced locomotor activity in female guinea pigs compared to males—bringing activity down to levels observed in males treated with cocaine alone (Wang et al., 2011). Although it's difficult to reconcile the findings described above, with one study reporting KOR-mediated increases in cocaine effects (Sershen et al., 1998) and the other study reporting KOR-mediated decreases (Wang et al., 2011), the studies highlight the importance of species, drug, and experimental design in modulating KOR effects. Furthermore, these studies touch upon the important interactions of KORs with the mesolimbic dopamine system and are broadly consistent with studies showing greater evoked dopamine release in female compared to male nucleus accumbens (Walker et al., 2000). It is possible that enhanced dopamine function can protect females from the inhibitory effects of KOR activation.

### Neuroanatomy of KORs related to affective state

In mammals, KORs are expressed throughout brain regions involved in affect, cognition, and motivated behavior, including the ventral tegmental area (VTA), nucleus accumbens (NAc), prefrontal cortex (PfC), hippocampus, dorsal striatum, amygdala, BNST, locus coeruleus, substantia nigra, dorsal raphe nucleus, pedunculpontine nucleus, and hypothalamus (Mansour et al., 1994a,b, 1995, 1996; Peckys and Landwehrmeyer, 1999; Svingos et al., 1999; Margolis et al., 2003; Le Merrer et al., 2009). The effect of KOR activation on neural transmission depends on the phenotype (e.g., excitatory or inhibitory) of the neuron expressing KORs, its projection target, and whether the receptor is expressed pre- or post-synaptically. These criteria have been elegantly described for KOR-mediated modulation of VTA dopamine neurotransmission. Broadly, KOR activation inhibits dopamine release (Margolis et al., 2003, 2005, 2006). The VTA sends dopaminergic efferents to many brain regions including the NAc, PfC, and basolateral amygdala. VTA dopamine neurons express KOR mRNA and the final protein expression patterns appear to depend on projection target (Ford et al., 2006; Margolis et al., 2006). Based on retrograde tracing from VTA target sites and electrophysiological recordings of VTA dopamine neurons (Margolis et al., 2006), it has been demonstrated that VTA neurons that project to the NAc express KORs predominantly on their nerve terminals within the NAc. In contrast, VTA dopamine neurons that project to the PfC express KORs predominantly on their cell bodies within the VTA. Consistent with this, administration of KOR agonists into the NAc decreases local dopamine concentrations (Donzanti et al., 1992; Spanagel et al., 1992), most likely by stimulating presynaptic KORs on dopaminergic afferents from VTA neurons (Svingos et al., 1999). In a separate study using similar techniques (Ford et al., 2006), it was shown that KOR activation inhibits VTA dopamine neurons projecting to the NAc but has little effect on dopamine neurons projecting to the basolateral amygdala. Furthermore, KOR activation selectively inhibits somatodendritic dopamine release (via KORs expressed on dopamine neuron cell bodies) in NAc projection neurons. DYN can modulate VTA dopaminergic neurons via actions on non-dopamine neurons as well. For example, KORs are likely expressed on GABAergic (Ford et al., 2006; Graziane et al., 2013; Crowley and Kash, 2015) and glutamatergic (Margolis et al., 2005) neurons that synapse on dopamine neurons in the VTA. Indeed, DYN has been shown to inhibit glutamate transmission in the VTA (Mu et al., 2011). The origin of DYN inputs to the VTA is not fully understood, but comes in part from the nucleus accumbens and lateral hypothalamic neurons that co-express orexin (Muschamp et al., 2014).

KORs and DYN are also enriched in the basolateral amygdala, the central nucleus of amygdala, and the BNST (Crowley and Kash, 2015). In the central nucleus of amygdala, pDYN mRNA is expressed in at least some CRF-expressing neurons (Marchant et al., 2007). The highest concentration of KOR binding in the rodent is found in the endopiriform cortex (Le Merrer et al., 2009). The endopiriform cortex is relatively understudied but appears to be an area of convergence for sensory and affect-related information and is involved in aspects of the storage, consolidation, and retrieval of emotional memories (de Curtis and Paré, 2004). In the guinea pig endopiriform cortex, KOR levels are higher in males compared to females (Wang et al., 2011), but the functional significance of this is unkown.

In the hippocampus, DYN is localized in dentate gyrus granule cells, from which it is locally released to act as a retrograde transmitter inhibiting excitatory inputs within the hippocampus (Drake et al., 1994). The dentate gyrus granule cells form the mossy fiber pathway that is involved in learning related to drug abuse (Torres-Reveron et al., 2009). DYN and ERβ, but not ERα, are colocalized in the mossy fiber pathway. Female rats in estrus have been shown to have increased DYN levels in the dentate gyrus and certain CA3 lamina compared to rats in proestrus or diestrus (Torres-Reveron et al., 2009). Also, U50,488-stimulated [^35^S]GTPγS binding in the dentate gyrus of guinea pigs has been shown to be higher in females compared to males (Wang et al., 2011).

## Mechanisms underlying sex differences in DYN/KOR function

### Polymorphisms in the prodynorphin gene

Both environmental and genetic factors significantly increase vulnerability to drug addiction (Tsuang et al., 1996; Clarke et al., 2012). Most genetic variation in humans is attributable to single nucleotide polymorphisms (SNPs). These are positions on a DNA sequence at which two alternative bases occur in at least 1% of the human population (Wang et al., 1998). The remaining genetic variation in humans is due to insertions or deletions of one or more nucleotides in genes, repeat length polymorphisms and DNA rearrangements (Sachidanandam et al., 2001). Sex-linked polymorphisms in the pDYN gene have been associated with increased vulnerability to develop drug addiction (Clarke et al., 2009). Specifically, two SNPs, rs1997794 in the promoter and rs1022563 in the 3′ UTR region of the pDYN gene have been shown to be significantly associated with opioid dependence in women but not men in a Chinese population, suggesting that these SNPs confer an increased risk for women (Clarke et al., 2009). In European Americans, SNPs rs1022563, rs910080, and rs199774 have been shown to be significantly associated with an increased risk for opioid addiction in both men and women. However, the odds ratio was higher in women compared to men (Clarke et al., 2012). In contrast, no significant associations with SNPs in the pDYN gene have been found in African Americans (Clarke et al., 2012). SNPs in the 3′ UTR of the pDYN gene (rs910080 and rs2235749) have been associated with decreased pDYN expression (Yuferov et al., 2009). This raises the possibility that DYN is decreased in women with pDYN SNPs that are associated with increased risk of developing opioid dependence. Future studies should determine why the association of these SNPs with opioid dependence is higher in women and also whether their presence can predict an increased risk of developing opioid use disorders in specific subpopulations of women.

### Sex-linked modulation of KOR analgesia by the melanocortin-1 receptor (MC1R) in females vs. the NMDA receptor in males

One of the most well characterized sex-linked mechanisms in KOR-mediated opioid analgesia is the selective involvement of N-methyl-D-aspartate receptors (NMDARs) in males vs. that of melanocortin-1 receptors (MC1Rs) in females (Mogil and Bailey, 2010). NMDAR antagonists can block stress- and KOR-induced analgesia in males but not females (Mogil et al., 1993; Kavaliers and Galea, 1995; Kavaliers and Choleris, 1997; Mogil and Belknap, 1997). In contrast, MC1Rs are necessary for KOR-mediated analgesia in females but not males (Mogil and Bailey, 2010). These differences are hormonally dependent since chronic estrogen and acute progesterone have been shown to act as a “switch”: female mice utilize the MC1R system under normal conditions or after hormone replacement following gonadectomy but switch to the NMDAR system following gonadectomy or estropause (a state of reproductive senescence; Sternberg et al., 2004a,b).

Using Quantitative Trait Locus (QTL) mapping, Mogil and colleagues demonstrated that a region of distal mouse chromosome 8 that contains the MC1R gene is linked with stress-induced analgesia in female, but not male mice. QTL mapping is a technique in which the coinheritance of continuous traits and polymorphic DNA markers such as microsatellite markers or SNPs are used in order to identify the chromosomal location of genes that are responsible for trait variability (Lander and Schork, 1994; Mogil et al., 2003). Specifically, Mogil and colleagues measured U50,488-induced analgesia in mice and then genotyped them for three microsatellite markers spanning a 24-cM region on distal chromosome 8. They demonstrated a significant linkage between genotype and U50,488-induced analgesia in females, but not males. The QTL accounted for 25% of overall trait variance (Mogil et al., 2003). Female mice inheriting two copies of an allele at microsatellite marker *D8Mit56* inherited from the DBA/2J background mouse strain, which is particularly sensitive to the analgesic effects of opioids (Belknap et al., 1990) displayed twice as much U50,488-induced analgesia compared to female mice inheriting two copies of an allele inherited from the C57/6J background mouse strain. The allelic status of male mice at this marker had no consequence (Mogil et al., 2003).

In humans, MC1R switches melanin synthesis from the red/yellow phaeomelanin pathway to the black eumelanin pathway in both skin and hair (Sturm et al., 2001). Individuals with red hair are homozygotes or compound heterozygotes at major MC1R variants (R151C, R160W, D294H), which are associated with loss of function and reduced cAMP production (Bastiaens et al., 2001) upon receptor activation with α-melanocyte stimulating hormone (α-MSH), an endogenous ligand at MC1Rs. Women with variants of the MC1R gene that are associated with red hair and fair skin (i.e., loss of function variants) have been shown to display significantly greater pentazocine-mediated analgesia than men with the same variants (Mogil et al., 2003). Although it is not known how MC1R interacts with KORs in a female-specific manner, both KORs and MC1Rs signal through cAMP pathways (Bastiaens et al., 2001) and are found in the periaqueductal gray (PAG) and locus coeruleus (LC), two brain regions important for sensory and affective dimensions of pain (Caruso et al., 2014). One possible mechanism involves receptor heterodimerization, although this has not been directly tested.

### Heterodimerization of MOR and KOR

Traditionally, GPCRs have been thought to act as monomers, but more recent research suggests that GPCRs may form homo or heterodimers as part of their normal trafficking and function (Prinster et al., 2005). As such, heterodimerization is an important mechanism underlying receptor function (Kern et al., 2012). In some cases heterodimerization may even be required for the surface expression and functionality of a receptor. Even if heterodimerization is not absolutely required, it can still result in dramatic changes in receptor pharmacology (i.e., increase specificity for a ligand), internalization processes (Prinster et al., 2005; Lohse, 2010), and it can stabilize specific receptor conformations that promote coupling with discrete downstream effectors (Kern et al., 2012).

**Figure 2:**
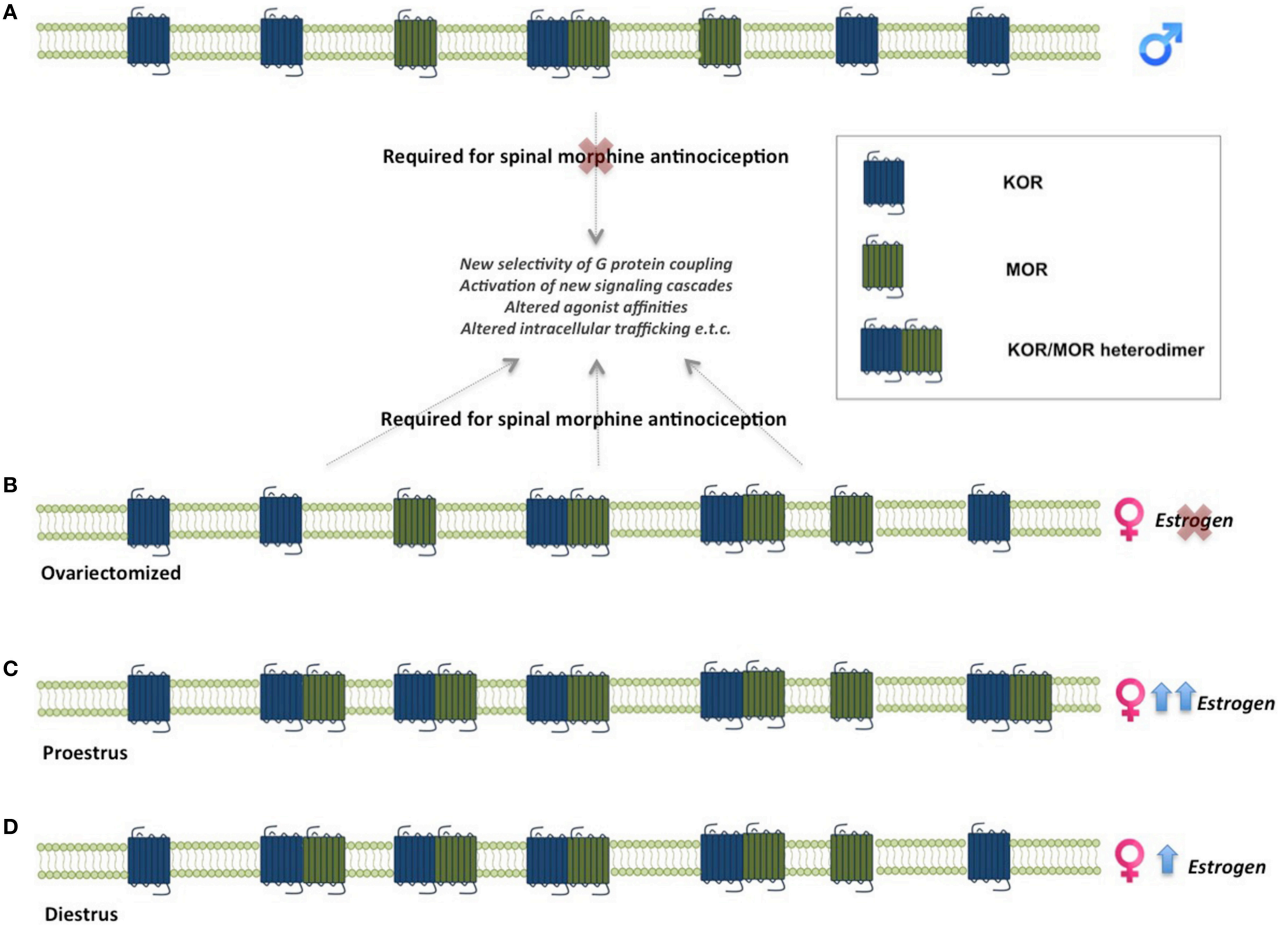
**Sex-linked heterodimerization of mu and kappa opioid receptors**. MORs and KORs are expressed in the spinal cord in both males and females. In males, morphine-mediated analgesia does not require concomitant activation of these receptors, whereas activation of MOR/KOR heterodimers in females is necessary for morphine analgesia. **(A)**. MOR/KOR heterodimers are more highly expressed in ovariectomized females compare to males **(B)**. Estrous cycle stage and estrogen levels affect the formation of heterodimers in the female spinal cord. Levels of MOR/KOR heterodimers are higher during proestrus **(C)** compared to diestrus **(D)**, suggesting an estrogen-dependent mechanism underlies their formation (Chakrabarti et al., 2010; Liu et al., 2011). KOR, kappa opioid receptor; MOR, mu opioid receptor.

One of the mechanisms suggested to underlie sex differences in the antinociceptive effects of opioids is the formation of MOR and KOR heterodimers (Chakrabarti et al., 2010). MOR/KOR heterodimers have been shown to be most prevalent in the spinal cord of proestrous females (cycle stage with highest estrogen levels), followed by females in diestrous and estrous and finally in males (Chakrabarti et al., 2010), suggesting that estrogen contributes to their formation (Figure 2). Consistent with this, spinal synthesis of estrogen and subsequent rapid signaling through membrane-localized ERs regulate MOR/KOR heterodimerization (Liu et al., 2011). Blockade of either ERα, ERβ or progesterone receptor (PR) substantially reduces MOR/KOR heterodimers and shifts morphine-induced antinociception from being KOR-dependent to KOR-independent (Liu et al., 2011). Exogenous or endogenous estrogen enhances KOR-mediated antinociception in females, whereas testosterone has no effect in males (Lawson et al., 2010). Despite the clear regulation by estrogen, it has also been reported that MOR/KOR heterodimers are more prevalent in ovariectomized females compared to males (Chakrabarti et al., 2010) indicating an additional sex-dependent but estrogen independent mechanism. MOR/KOR heterodimers have been shown to utilize spinal DYN_1−17_ as a substrate for the female-specific KOR component of spinal morphine antinociception (Chakrabarti et al., 2010).

To date, MOR and KOR heterodimerization has not been described in neural circuits that modulate motivated behavior. The necessary ingredients are present, as there is substantial overlap in MOR and KOR expression in brain regions such as the NAc, BNST, PfC, VTA, and amygdala (Le Merrer et al., 2009). Furthermore, ERα and ERβ are also expressed in these regions (Figure 2), which would allow for estrogen-mediated facilitation of MOR/KOR heterodimers, as described above. The functional consequences of putative MOR/KOR heterodimers within reward circuits are not easy to conceptualize, as behavioral effects of MOR and KOR agonists tend to oppose each other. This is different from pain systems, in which both MOR and KOR agonists have analgesic properties. A recent study demonstrated heterodimerization of orexin receptor 1 and KORs in the VTA (Chen et al., 2015), which is consistent with the idea that MOR/KOR heterodimers in similar brain regions are feasible.

### Gonadal hormone mechanisms of KOR function

There is substantial evidence for organizational and activational roles of gonadal hormones in mediating effects of KORs (Forman et al., 1989; Limonta et al., 1989; Mogil et al., 1993; Rasakham and Liu-Chen, 2011), including roles in the processes described above. However, the actual mechanisms through which hormones mediate their effects are not fully understood. It is also important to note that DYN and KOR function can be sex-dependent but hormone-independent. For example, it has been shown in the four-core genotype mouse model that XX mice that have either male or female gonads have higher pDYN mRNA expression in the striatum compared to XY mice that have either male or female gonads (Chen et al., 2009). This is likely due to incomplete inactivation of portions of the X chromosome.

The most straightforward manner in which gonadal steroids might influence KOR function is via transcriptional mechanisms involving steroid hormone receptor binding to estrogen or androgen response elements found in regulatory regions on genes (Gruber et al., 2004; Klinge et al., 2004; Gottsch et al., 2009). As an example, estradiol has been shown to decrease DYN expression in the arcuate nucleus of the hypothalamus in wild-type but not ERα-/- mice through an ERE-dependent pathway (Gottsch et al., 2009). These findings suggest that DYN-expressing neurons in the arcuate play a role in estrogen-mediated negative feedback of pulsatile gonadotropin-releasing hormone (GnRH)/luteinizing hormone (LH) release (Mostari et al., 2013). There is also evidence that testosterone can regulate DYN expression. In castrated Romney Marsh rams it has been shown that testosterone treatment increases pDYN mRNA in the supraoptic nucleus and the BNST during the breeding season (Scott et al., 2008).

A more indirect mechanism for gonadal hormone control of gene expression is through modulation of transcription factor activity. For example, the pDYN gene contains cAMP response element (CRE) sequences in its promoter that bind the transcription factor CREB (Douglass et al., 1994; Cole et al., 1995). Estrogen treatment has been shown to increase CREB phosphorylation in the anteroventral periventricular nucleus, resulting in elevated pCREB in approximately 25% of pDYN-positive neurons in this region (Gu et al., 1996).

Gonadal hormones can also influence DYN and KOR function through rapid effects at membrane receptors and non-transcriptional effects on second messenger pathways. Pain tolerance is increased during pregnancy (Cogan and Spinnato, 1986), an effect due in part to enhanced KOR-mediated antinociception in the spinal cord (Gupta et al., 2001; Gintzler et al., 2008). This phenomenon of gestational antinociception can be mimicked in gonadectomized rodents by administering pregnancy level concentrations of estrogen and progesterone (Dawson-Basoa and Gintzler, 1998; Gintzler and Liu, 2012). It has been shown that ERα is coexpressed with DYN in the dorsal horn of the spinal cord, and this coexpression is increased during pregnancy or after estrogen and progesterone treatment (Gintzler et al., 2008). ERα activation contributes to increased DYN release in the spinal cord, possibly through ERα-mediated activation of PKA and MAPK pathways (Gintzler et al., 2008). An alternate, but not mutually exclusive, possibility is that membrane bound ERs increase neuronal excitability through activation of metabotropic glutamate receptors (Boulware et al., 2005). In addition to coexpression of ERα and DYN, delta opioid receptors (DORs) are expressed on DYN terminals in the dorsal horn. Under normal (non-pregnant) conditions, DOR activation inhibits DYN release in the spinal cord and attenuates KOR-mediated analgesia. However, during pregnancy, this effect is switched such that DOR activation stimulates DYN release and facilitates gestational antinociception. Although the mechanisms underlying this switch in DOR function are not understood, it is likely mediated by effects of ER activation on intracellular signaling events that alter DOR G-protein coupling from inhibitory to excitatory (Gintzler et al., 2008).

## Applying mechanisms of sex differences in KOR/DYN systems from pain and other systems to drug addiction

There are at least two ways in which mechanistic studies of sex differences in KOR/DYN systems within pain circuits can apply to drug addiction. First, many of the brain regions subserving pain are also important for reward and affective states (see Figure 1), raising the possibility that sex differences in pain modulation—particularly the affective component of pain—also hold true for sex differences in addiction. As one example, MC1Rs and KORs are colocalized in the periaqueductal gray (PAG) and locus coeruleus (LC), two brain regions important for sensory and affective dimensions of pain (Caruso et al., 2014) as well as somatic and affective dimensions of opiate withdrawal (Williams et al., 2001). There is evidence that KOR activation is necessary for morphine withdrawal-induced conditioned place aversions (Kelsey et al., 2015). Although not yet studied, it is possible that KOR-mediated aversive effects of morphine withdrawal in females depends on MC1Rs. The second way in which mechanisms underlying sex differences in KOR function can apply to addiction is that molecular and biochemical processes are typically similar across brain regions. What makes the functional difference is the neural circuit within which the processes are found. As one example, sex-linked polymorphisms in the pDYN gene will result in alterations in DYN in all cells. The effect these alterations have will depend on the particular cell type and brain region studied.

## Future directions

Major technical advances in genetics, molecular biology, and neurophysiology have enabled the discovery of genes, cellular mechanisms and neural circuits necessary for the transition from drug use to drug addiction. Most of these advances have been made selectively in male animal models, but fortunately more and more research is being conducted on males and females in parallel. In our view, it is essential to keep in mind that sex differences occur at multiple levels (McCarthy and Arnold, 2011). Of particular importance to this review, sex differences can manifest as behavioral differences in which the average behavior is different in males and females. This type of sex difference is the most commonly reported and includes the discussed differences in pain thresholds, analgesic efficacy of KOR ligands, and aversive effects of KOR activation, to name a few. These behavioral differences suggest that there are distinct underlying molecular mechanisms between males and females. Another level at which sex differences can occur is when behavioral endpoints are the same in males and females, but underlying neural mechanisms differ. This type of sex difference is often missed since there is no overt sex difference in behavior. An example of this is the female requirement for MC1R activation but male requirement for NMDA receptor activation in KOR-mediated analgesia.

A primary goal of this review was to synthesize what is known about sex differences in DYN and KOR systems and relate this to drug addiction. At this point, very little is known that directly connects sex differences in DYN and KOR function with sex differences in drug addiction. We identify three major gaps in knowledge:

1. Are there sex differences in how DYN and KORs contribute to different facets of addictive behavior (e.g., acquisition, maintenance, dependence, withdrawal, craving, and relapse)?
2. Are there sex differences in DYN and KOR expression levels and patterns, DYN release, and KOR coupling to downstream effectors within neural circuits that modulate addictive behavior (e.g., mesocorticolimbic system, extended amygdala, hypothalamic pituitary adrenal [HPA] axis)?
3. Is the function of DYN and KORs within these addiction-related neural circuits dependent on gonadal hormones? If so, what are the mechanisms?

Based on genetic association studies of the pDYN gene in drug-dependent populations (Clarke et al., 2009; Yuferov et al., 2009) and the known mechanisms underlying KOR function in antinociception described in the preceding section, we propose and encourage similar studies in animal models of drug addiction. For example, is there evidence for KOR/MOR heterodimers in limbic brain circuits? Are either MC1R or NMDA receptors necessary for KOR-mediated effects on motivated behavior? Can steroid hormone receptors modulate transcription of DYN or KOR or modify KOR-mediated intracellular signaling pathways in limbic circuits? Given the impact of negative affect on the maintenance of addiction, delineating the underlying mechanisms in both males and females will allow the development of treatments that are maximally effective in each sex.

## Author contributions

EC and MM contributed equally to the literature research, organizing, writing, and editing of this review article.

## Funding

This work was supported by the National Institute on Drug Abuse grant DA033526 (to EC) and the Jonathan E Brooking Fellowship (to MM).

### Conflict of interest statement

The authors declare that the research was conducted in the absence of any commercial or financial relationships that could be construed as a potential conflict of interest.
